# Morphological diversification of biomechanical traits: mustelid locomotor specializations and the macroevolution of long bone cross-sectional morphology

**DOI:** 10.1186/s12862-019-1349-8

**Published:** 2019-01-30

**Authors:** Brandon M. Kilbourne, John R. Hutchinson

**Affiliations:** 1 Museum für Naturkunde Berlin, Leibniz Institut für Evolutions- und Biodiversitätsforschung, Invalidenstraße 43, 10115 Berlin, Germany; 2 Structure and Motion Laboratory, Department of Comparative Biomedical Sciences, The Royal Veterinary College, Hawkshead Lane, Hatfield, AL9 7TA UK

**Keywords:** Mustelidae, Locomotion, Trait evolution, Adaptation, Morphological diversification, Cross-sectional properties

## Abstract

**Background:**

Morphological diversity of limb bone lengths, diameters, and proportions in mammals is known to vary strongly with locomotor habit. It remains less well known how different locomotor habits are correlated with cross-sectional traits of the limb skeleton, such as cross-sectional area (CSA), second moments of area (SMA), and section modulus (MOD) and whether these traits have evolved adaptively. CSA and SMA represent the bone’s resistance to axial compression and bending, respectively, whereas MOD represents bone structural strength related to shape. Sampling 28 species of mustelids, a carnivoran lineage with diverse locomotor habits, we tested for differences in humeral, radial, and ulnar cross-sectional traits among specialists for climbing, digging, and swimming, in addition to generalists. Given that the limbs of digging specialists function in the dense substance of soil, and that swimming specialists need to counteract buoyancy, we predicted that these mustelids with these specializations should have the greatest values of cross-sectional traits.

**Results:**

We analyzed cross-sectional traits (calculated via μCT scanning and rendered dimensionless) in 5% increments along a bone’s length and found significant differences among locomotor habits, though differences in ulnar cross-sectional traits were fewer than those for the humerus and radius. Swimming specialists had the greatest values of cross-sectional traits, followed by digging specialists. Climbing specialists had the lowest values of cross-sectional traits. However, phylogenetic affinity underlies these results. Fitting models of trait evolution to CSA and SMA revealed that a multi-rate Brownian motion model and a multi-optima Ornstein-Uhlenbeck model are the best-fitting models of evolution for these traits. However, inspection of α-values uncovered that many of the OU models did not differ from a Brownian motion model.

**Conclusions:**

Within Mustelidae, differences in limb function and locomotor habit influence cross-sectional traits in ways that produce patterns that may diverge from adaptive patterns exhibited by external traits (e.g., bone lengths) of the mammalian limb skeleton. These results suggest that not all the traits of a single organ evolve under a single evolutionary process and that models of trait evolution should be fit to a range of traits for a better understanding of the evolution of the mammalian locomotor system.

**Electronic supplementary material:**

The online version of this article (10.1186/s12862-019-1349-8) contains supplementary material, which is available to authorized users.

## Introduction

Specializations in mammalian limb morphology are well documented and have been the subject of study for more than a century [21, 33, 66, 75, 91], with these studies primarily focusing on bone lengths, diameters, and the in-levers of muscles [9, 10, 12, 23, 27, 28, 39, 40, 53, 69, 78, 84, 87, 88, 97]. Notably, differing locomotor habits within mammals are associated with distinct limb morphologies. Scansorial/climbing mammals are characterized by limb skeletons with relatively elongate and gracile elements both proximally and distally, including elongate digits, whereas cursorial/running mammals are characterized by limb skeletons with gracile elements and elongate distal limb elements. In contrast, fossorial/digging mammals are characterized by more robust and relatively shorter long bones (for shorter muscle out-levers) and longer muscle in-levers (e.g., olecranon process), some of which exhibit large tuberosities (e.g., the deltoid ridge/tuberosity). Natatorial/swimming mammals are also characterized by robust long bones, longer olecranon processes, and, in species relying upon the hindlimb for swimming, elongate metatarsals and phalanges. Remarkably, locomotor adaptations similar to these traits are also known to occur in early mammaliaform taxa with regards to digging (e.g., [67]), swimming (e.g., [49]), and climbing (e.g., [70]).

**Fig. 1:**
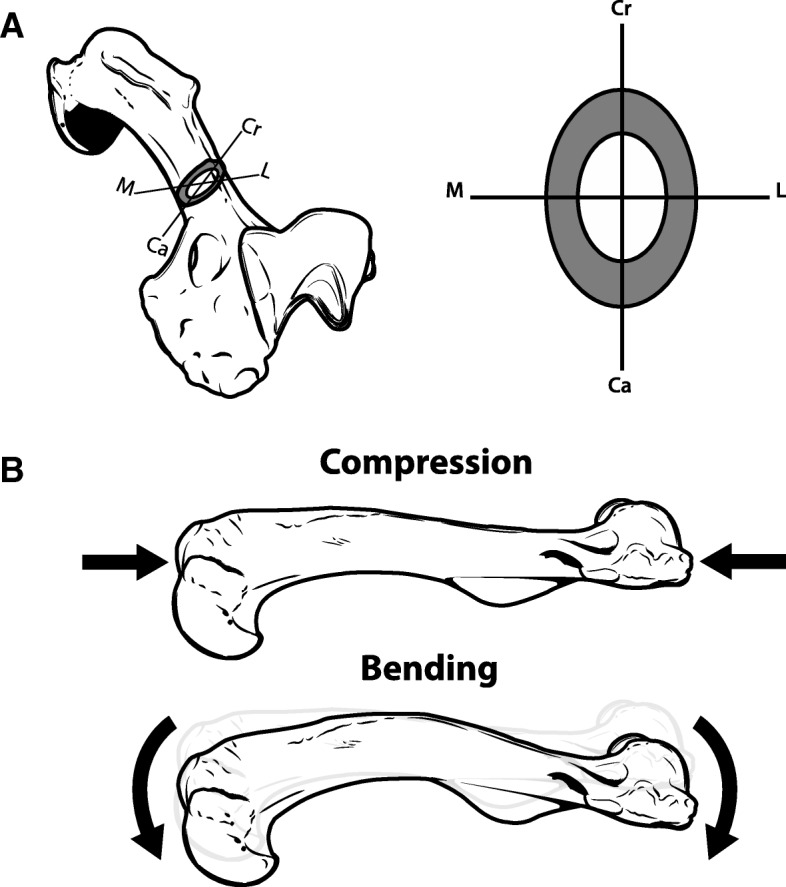
Cross-sectional traits and example loading regimes. In A, a humerus showing the orientation of anatomical axes about which SMA was measured: cranio-caudal (Cr-Ca) and medio-lateral (M-L). The total bone tissue (dark grey) within the cross-section determines the bone’s cross-sectional area (CSA), whereas the distribution of bone tissue about specified axes determines the bone’s second moment of area (SMA). CSA and SMA withstand specific forms of mechanical loads (B). CSA determines resistance to forces causing axial compression along the length of the bone, whereas SMA determines resistance to bending moments that cause a bone to flex about a given axis. SMA_ML_ is associated with bending about the M-L axis (i.e., bending in the Cr-Ca plane), whereas SMA_CC_ is associated with bending about the Cr-Ca axis (i.e., bending in the M-L plane). Notably the farther bone tissue is from an axis of bending, the greater the resistance to bending. Thus in A, SMA_ML_ is greater than SMA_CC_. Note that the example of bending in B is exaggerated for illustrative purposes, and all cross-sectional traits were measured with BoneJ 1.4.2 [17]

In spite of the extensive work on adaptations of the external traits of the limb skeleton, particularly long bones, there has been noticeably less investigation into adaptations of traits associated with the internal geometry of long bones: cross-sectional area (CSA), second moment of area (SMA), and section modulus (MOD) (Fig. 1). CSA represents resistance to axial compression, whereas SMA represents resistance to bending about a specified axis. MOD represents structural strength due to cross-sectional shape, with higher values representing a bone being able to incur greater maximum bending moments relative to maximum mechanical stress. Adaptive evolution should occur among these traits, as differing locomotor habits most likely entail differing mechanical demands acting upon the limb skeleton [13, 39, 40, 78, 87, 88, 101]. For instance, the functioning of fossorial limbs in soil should be associated with higher values of CSA, SMA, and MOD due to the high density of soil (1.83–2.58 g/cm^3^; [85]). Notably, limbs specialized for digging have longer in-levers for stronger joint extension and often long bones with more robust external dimensions [22, 23, 53, 78, 84]. Similarly, the limbs of natatorial taxa are also associated with a greater robustness and more prominent in-levers [9, 78], and greater values of bone cross-sectional traits could also be beneficial to mitigating larger mechanical loads due to functioning in water, which also has a high density. However, a likely more critical biomechanical factor for natatorial tetrapods is the need to counteract buoyancy in aquatic environments. Among natatorial tetrapods, increased long bone cortical thickness and/or compactness has been concluded to counteract buoyancy during diving and aquatic foraging [5, 41–43], and greater cortical thickness and compactness should be reflected by greater CSA values, if not SMA and MOD values as well.

Testing for the potential for adaptations in bone cross-sectional traits in mammals requires a lineage that exhibits a range of locomotor behaviors. Mustelids are a species-rich lineage of carnivoran mammals that include scansorial, fossorial, and natatorial specialists, in addition to a more generalized locomotor habit. Within mustelids, the forelimbs play a role in each of their exhibited locomotor habits, including climbing [24, 27, 38], digging [71, 84], and swimming [9, 30], suggesting that the long bones of the forelimb are ideal for determining if divergent locomotor habits are associated with differences in bone cross-sectional traits. Here we test whether CSA, SMA, and MOD of the humerus, radius, and ulna differ (for a given body size) among fossorial, natatorial, scansorial, and generalized mustelids to understand biomechanical differences among mustelids of differing locomotor habits. More specifically, we predict that fossorial and natatorial mustelids will have the highest values of cross-sectional traits. Regarding fossorial mustelids, this prediction is due to their limbs functioning in soil, which is highly dense (1.83–2.58 g/cm^3^; [85]) and thus likely subjects a limb engaged in digging to high mechanical loads. Regarding natatorial mustelids, this prediction stems from the moderately high density of water (1.0 g/cm^3^), which could subject a limb engaged in swimming to higher mechanical loads, and the additional need for natatorial taxa to counteract buoyancy in aquatic environments. We also predict that forelimb cross-sectional traits have evolved adaptively within mustelids under selective regimes relating to limb function, and here fit competing models of trait diversification to test this.

## Materials and methods

**Fig. 2:**
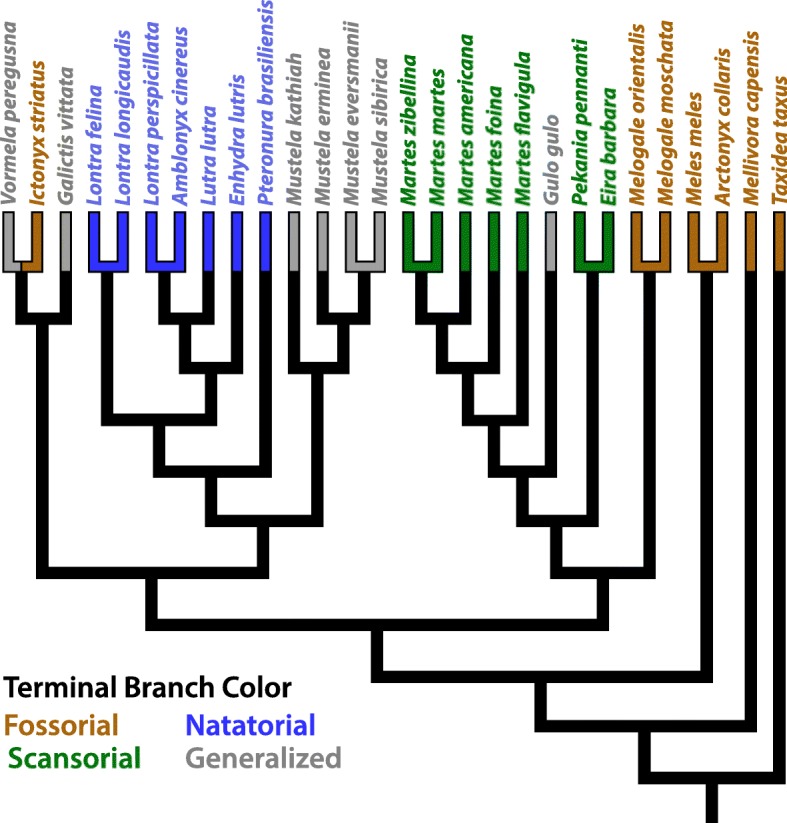
Phylogeny of the sampled Mustelidae. The color of terminal branches and taxon names indicate the locomotor habit for each mustelid species. To obtain this phylogeny, the phylogeny of Law et al. [63] was pruned to our sampled taxa

Cross-sectional traits were measured from x-ray computed tomography (CT) scans of the humerus, radius, and ulna at 5% increments along the length of each bone (Fig. 1) in 28 species of mustelid (Table 1; Fig. 2). To minimize the influence of differences in sample size among mustelid locomotor habits, we sampled seven taxa each from generalized, fossorial, natatorial, and scansorial mustelids. All sampled individuals were adults, as determined by the epiphyses being fully fused to the metaphyses. The only exception to this is the specimen representing *Melogale orientalis*, the Javan ferret badger (ZMB MAM 8949). Although it was a subadult, we chose to sample this specimen, as postcranial material for *Melogale sp*. is rare in museum collections.

**Table 1:** Scanned mustelid species alongside their locomotor habit. N = number of specimens for that species. Scan Location indicates the facility where CT scans were made: 1) Museum für Naturkunde Berlin, Berlin, Germany, 2) Royal Veterinary College, Hertfordshire, UK and 3) University of Chicago, Chicago, USA

Species	N	Common Name	Habit	Reference	Scan Location
*Amblonyx cinereus*	2	Asian small-clawed otter	Natatorial	Larivière [60]	1
*Arctonyx collaris*	1	Hog badger	Fossorial	Nowak [73]	1
*Eira barbara*	2	Tayra	Scansorial	Presley [80]	1
*Enhydra lutris*	1	Sea otter	Natatorial	Estes [26]	1
*Galictis vittata*	1	Greater grison	Generalized	Yensen & Tarifa [102]	3
*Gulo gulo*	1	Wolverine	Generalized	Pasitschniak-Arts & Larivière [76]	1,2
*Ictonyx striatus*	2	Zorilla	Fossorial	Larivière [59]	1
*Lontra felina*	1	Marine otter	Natatorial	Larivière [57]	1
*Lontra longicaudis*	1	Long-tailed otter	Natatorial	Larivière [58]	1
*Lutra lutra*	2	Eurasian otter	Natatorial	Hung & Law [46]	1
*Lutrogale perspicillata*	1	Smooth-coated otter	Natatorial	Hwang & Larivière [48]	3
*Martes americana*	2	N. American marten	Scansorial	Clark et al. [14]	3
*Martes flavigula*	2	Yellow-throated marten	Scansorial	Larivière & Jennings [61]	1
*Martes foina*	1	Beech marten	Scansorial	Larivière & Jennings [61]	1
*Martes martes*	2	Pine marten	Scansorial	Larivière & Jennings [61]	1
*Martes zibellina*	2	Sable	Scansorial	Larivière & Jennings [61]	1
*Meles meles*	2	European badger	Fossorial	Larivière & Jennings [61]	1
*Mellivora capensis*	1	Honey badger	Fossorial	Vanderhaar & Hwang [98]	1
*Melogale moschata*	1	Chinese ferret-badger	Fossorial	Storz & Wozencraft [95]	1
*Melogale orientalis*	1	Javan ferret-badger	Fossorial	Nowak [73]	1
*Mustela erminea*	2	Ermine	Generalized	King [54]	1
*Mustela eversmanii*	1	Steppe polecat	Generalized	Larivière & Jennings [61]	1
*Mustela kathiah*	1	Yellow-bellied weasel	Generalized	Larivière & Jennings [61]	1
*Mustela sibirica*	1	Siberian weasel	Generalized	Law [62]	1
*Pekania pennanti*	1	Fisher	Scansorial	Powell [79]	1
*Pteronura brasiliensis*	1	Giant otter	Natatorial	Noonan et al. [72]	1
*Taxidea taxus*	2	N. American badger	Fossorial	Long [65]	1
*Vormela peregusna*	1	Marbled polecat	Generalized	Gorsuch & Larivière [32]	1

CT scans were generated at the Museum für Naturkunde Berlin, the University of Chicago, and the Royal Veterinary College (Table 1). µCT scans were made on a Phoenix|x-ray Nanotom (GE Sensing and Inspection Technologies GmbH, Wunstorf, Germany) in Berlin and a Phoenix|x-ray Nanotom and v|tome|x combination in Chicago, respectively. At the Royal Veterinary College, lower resolution medical CT scans were made on a GE LightSpeed 16 scanner (GE Medical Systems, Pollards Wood, UK). X-ray slices were reconstructed using the software datos|x-reconstruction version 1.5.0.22 (GE Sensing and Inspection Technologies GmbH, Phoenix|x-ray) and resulting reconstructed bones were oriented in VG Studio Max 2.0 and 2.1 (Volume Graphics, Heidelberg, Germany).

For each μCT scan, DICOM image stacks of > 1000 slices were generated sequentially along a bone’s proximo-distal axis, whereas for medical quality scans, image stacks of 250–270 slices were generated. For each image stack, the number of the slices containing the proximal-most and distal-most extremes of an individual bone were identified. To obtain slices at 5% increments along the length of the bone, the difference was calculated between the slice numbers containing the proximal and distal extremes of a bone, and this value was divided by 20 to downsample the data to 21 slices. Slices analyzed for cross-sectional traits were segmented in ImageJ 1.51n [90]. For segmentation, the area of the image external to the perimeter of the bone was replaced with black pixels (greyscale value = 0) by using the freehand selection tool in combination with the Fill and Clear Outside commands. After segmentation, the 16-bit images produced by VG Studio were converted into 8-bit prior to data collection with BoneJ 1.4.2 [17]. Cross-sectional properties were then collected using BoneJ’s slice geometry function with an orientation of the cranio-caudal axis set to 270°. The minimum greyscale value associated with bone tissue was manually determined for each segmented slice, whereas the maximum greyscale value associated with bone tissues was 255, as all possible background noise and image artifacts had been previously removed with segmentation.

Using BoneJ, the following traits were measured: CSA, SMA, and MOD. In addition to their biomechanical relevance, CSA represents the total amount of bone tissue in a cross-section, whereas SMA and MOD represent the distribution of the total amount of bone tissue about specified axes. We chose two anatomical axes with regards to measuring SMA and MOD: cranio-caudal (CC) and medio-lateral (ML) (Fig. 1). With bone tissue being identified by maximum and minimum greyscale values, and pixels calibrated to mm within the DICOM image format, BoneJ calculated a bone’s CSA from pixels falling in the appropriate range of greyscale values. SMA is calculated as SMA = Σ(A⋅d^2^), where *A* is an unit of area and *d* is the distance of that unit from the cross-section’s neutral axis, and MOD is calculated as MOD = SMA/c, where *c* is the distance from the neutral axis to the farthest unit of bone tissue [7]. BoneJ calculates SMA and MOD using the greyscale values denoting bone tissue and pixel coordinates [17]. Given a difference in size of  two orders of magnitude exists in our sample (e.g., *Mustela kathiah* [0.21 kg] vs. *Enhydra lutra* [29.50 kg]; [63]), we rendered the trait values dimensionless as follows to facilitate comparison. We first reduced each trait to a single linear dimension, as CSA, SMA, and MOD have units of mm^2^, mm^4^, and mm^3^, respectively. We took the second root of CSA (i.e., CSA^1/2^), the fourth root of SMA (i.e., SMA^1/4^), and the third root of MOD (i.e., MOD^1/3^) to transform each of these traits into units of mm^1^. After reducing each trait to a single linear dimension, the trait value was further divided by the bone’s proximodistal (inter-articular) length to render it dimensionless. In addition to these traits, we also calculated a dimensionless ratio characterizing a bone’s relative resistance to bending vs. compression:

$$ R=\frac{SMA}{CSA} $$

This metric uses dimensionless SMA and dimensionless CSA and represents the ratio of the resistance to bending (i.e., SMA) to resistance to axial compression (i.e., CSA). This ratio was calculated separately for SMA_ML_ (i.e., R_ML_) and SMA_CC_ (i.e., R_CC_). Comparison of this metric across locomotor habits can reveal whether particular locomotor habits are associated with long bones more predisposed to withstanding bending vs. compression.

### Statistical analyses

The study of the evolution of biomechanical traits analytically requires a dual approach: an ahistorical approach focused on how the diversity of observed trait values relates to differences in biomechanical capability among species and a historical approach focused on how phylogeny influences biomechanical trait diversity and the underlying processes governing the evolution of these traits. An ahistorical approach is necessary as the mechanics imposed by a species’ ecological niche can only act upon the trait values possessed by the species and not upon a ‘phylogenetically corrected’ trait value. For instance, a bone’s internal stress due to compression (σ_compression_) would stem from a compressive force (F) acting on that bone’s CSA ((σ_compression_ = F/CSA). Likewise, a bone’s internal stress due to bending (σ_bending_) would stem from a bending moment (M), the bone’s SMA, and an additional term, y, which denotes the furthest distance of bone tissue from the bone’s neutral axis (σ_bending_ = My/SMA). These two calculations of bone stress apply regardless of the specific evolutionary processes underlying trait values. Species must be capable of meeting the biomechanical demands of their ecological niche, so an ahistorical analysis would also be informative for understanding how the biomechanical demands of species’ occupied niches are reflected in morphology.

However, as raised by Felsenstein [29], ahistorical analyses fail to address the influence of the shared ancestry of species upon observed trait values. Likewise, while an ahistorical analysis may contribute to the development of hypotheses regarding specific evolutionary processes (e.g., natural selection), they can in no way directly test for the past action of specific evolutionary processes upon trait diversification. To understand the role of phylogenetic non-independence in trait diversity, as well as to discern likely processes responsible for trait diversification, phylogenetic comparative methods must be employed to put biomechanical traits in a macroevolutionary context.

To address whether the differing biomechanical demands of fossorial, scansorial, natatorial, and generalist locomotor habits may be reflected in cross-sectional morphology, we tested for differences in cross-sectional traits by performing standard one-way ANOVAs using Tukey’s posthoc test (*P*_significance_ ≤ 0.05) for each separate trait, with locomotor habit being the independent factor. Additionally, we tested for differences in cross-sectional traits at 5% increments along the bone’s length, resulting in 19 increments (5–95%). This allowed us to determine if the association between locomotor habit and cross-sectional traits varied along a bone’s length. As we were comparing cross-sectional traits for 19 increments along a bone’s length, we also performed Bonferroni corrections (*P*_Bonferroni_ = 0.05/19 = 0.0026). ANOVAs were performed in R vers. 3.3.1 [81]. To address the role of phylogenetic relatedness, we also performed phylogenetic ANOVAs following the methodology of Adams & Collyer [2] by using the R package geomorph [3]. We additionally assess the clustering of locomotor habit within mustelid phylogeny using two-block partial least squares to test for a correlation between mustelid phylogeny and locomotor habit [2].

**Fig. 3:**
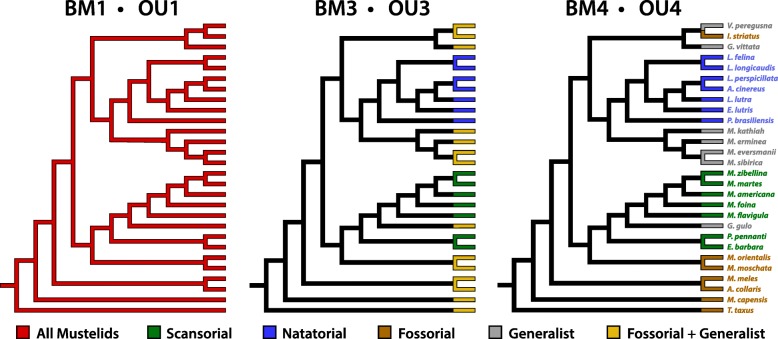
Three hypothetical models of the evolution of cross-sectional morphology in Mustelidae. The phylogeny in A represents a single rate (BM1) or single phenotypic optimum (OU1) process acting across all branches of the phylogeny, both internal and external. The phylogeny in B distinguishes three distinct rates (BM3) or optima (OU3) acting at the terminal branches of the phylogeny, with natatorial, scansorial, and remaining mustelids (i.e., fossorial and generalist taxa) each having their rate/optimum. The phylogeny in C distinguishes four distinct rates (BM4) or optima (OU4) acting at the terminal branches of the phylogeny, one each for the four locomotor habits within Mustelidae. For the BM3/OU3 and BM4/OU4 models, rates and optima acting along internal branches were estimated using stochastic character mapping [8, 45], which reflects uncertainty in character states of internal branches (see main text)

To test whether differences in bone cross-sectional traits are likely due to adaptive evolution, we fitted three Ornstein-Uhlenbeck (OU) models of trait evolution to the cross-sectional traits for each 5% increment along a bone’s length: a single optimum (OU1), a three optima (OU3), and a four optima (OU4) model (Fig. 3). OU1 represents a single phenotypic optimum (which can roughly be thought of as a selective pressure) acting across all branches of the phylogeny, OU3 represents separate phenotypic optima for scansorial, natatorial, and remaining mustelids, and OU4 represents separate phenotypic optima for scansorial, natatorial, fossorial, and generalized mustelids. Additionally, for each OU model, we tested a counterpart Brownian motion (BM) model with distinct rates of BM evolution replacing OU phenotypic optima: a single rate (BM1), a three-rate (BM3), and a four-rate model (BM4). BM models were used because differing rates of evolution acting across the phylogeny can result in increased morphological disparity [74]. Lastly, we also fit an Early Burst (EB) model [36], a model in which the net rate of evolution decreases over time (or increases, depending on how the rate parameter is bounded). The purpose of fitting models (OU, BM, and EB) to each 5% increment measured was to determine if the evolutionary dynamics likely governing the evolution of cross-sectional morphology vary along a bone’s length. BM and OU models were fit in R using package OUwie [6], and the EB model was fit using the R package geiger [37]. The recently published phylogeny of Law et al. [63] (Fig. 2) was used for the fitting of trait evolution models, as well as to run our phylogenetic ANOVAs.

While locomotor habits are known for the terminal taxa in the phylogeny (Table 1), the locomotor habits along the internal branches of the phylogeny are not. To incorporate uncertainty in ancestral states of locomotor habits into our analyses, we performed stochastic character mapping [8, 45] with the R package phytools [82]. In this method, the discrete character in question is randomly mapped onto the internal branches of the phylogeny and the models are then fitted to the tree, with model parameters and criteria being calculated. This procedure of random mapping of the discrete character and model fitting is then repeated over a number of iterations (for the current study: 500), and the model parameters are then averaged over all iterations. Best-fitting models were determined for CSA and SMA at each 5% increment of a bone's length by calculating Akaike’s Information Criterion for small samples (AICc: [47]) and taking the mean of this parameter across all iterations. Using the mean AICc values, Akaike weights [100] were then calculated to determine the relative fit of each model. Akaike weights were calculated with the R package qpcR [94]. As both SMA and MOD both reflect the distribution of total bone tissue about an axis of bending, we chose to model the evolution of only one of these traits.

## Results

**Fig. 4:**
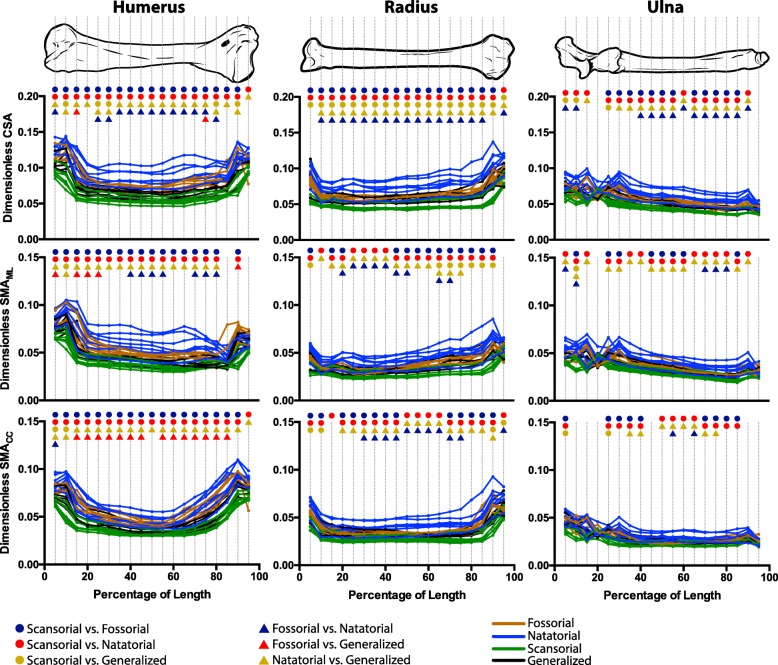
Differences in humeral, radial, and ulnar CSA and SMA among mustelid locomotor habits. Differences in CSA, SMA_ML_, and SMA_CC_ were tested at 5% increments along each bone’s length. CSA and SMA were rendered dimensionless by respectively taking the square and fourth root and dividing by bone length. Colored symbols indicate a significant difference (Adjusted *P* < 0.05) for the indicated pairwise comparison at a given increment. A lack of significant pairwise differences for a given increment indicates an overall ANOVA result of *P* > 0.0026 for that increment (the Bonferroni-corrected *P*-value). For the mechanical significance of these traits, see Fig. 1

For the humerus, radius, and ulna, long bone cross-sectional traits significantly differed among mustelid locomotor habits. Natatorial mustelids (e.g., otters) tended to have humeri with the greatest values of CSA, SMA (Fig. 4), and MOD (Additional file 1: Figure S1), followed by fossorial mustelids (e.g., badgers). Scansorial mustelids (e.g., martens) tended to have the lowest values. Notably across nearly the entire length of the humerus, natatorial and fossorial mustelids had significantly greater values of cross-sectional traits than scansorial mustelids. Additionally, natatorial mustelids had significantly greater values of cross-sectional traits than generalized mustelids (e.g., weasels) along largely the entire length of the humerus. Fossorial mustelids also had significantly greater SMA_CC_ and MOD_CC_ than generalist mustelids across almost the entire length of the humerus; significant differences in CSA, SMA_ML_, and MOD_ML_ between these two groups tended to be more localized along the length of the humerus. Primarily along the diaphysis, natatorial mustelids were found to have significantly greater CSA, SMA_ML_ and MOD_ML_ than fossorial mustelids. In contrast, significant differences in SMA_CC_ and MOD_CC_ were largely nonexistent between fossorial and natatorial mustelids. Notably, dimensionless trait values for the subadult *Melogale orientalis* did not appear to be outliers and clustered closely with other fossorial mustelids, in particular *Melogale moschata*.

Differences in cross-sectional traits of the radius were similar to those for the humerus. The most prevalent pairwise differences among locomotor groups were that natatorial mustelids significantly differed (i.e., greater values for traits) from both scansorial and generalized mustelids (Fig. 4 and Additional file 1: Figure S1). Additionally, natatorial mustelids significantly differed from fossorial mustelids with regards to CSA largely across the length of the radius; however, significant differences between these two locomotor habits were more regionalized to the diaphysis with regards to the remaining traits. Scansorial mustelids also exhibited significantly lower values of radial CSA than generalist taxa across the entire length of the radius.

Across the ulna’s length, significant differences in cross-sectional traits were recovered (Fig. 4 and Additional file 1: Figure S1). As was the case with the humerus and radius, the most ubiquitous pairwise differences consisted of natatorial mustelids having significantly greater values of cross-sectional traits than both scansorial and generalist mustelids. Natatorial mustelids also possessed greater values of cross-sectional traits than fossorial mustelids; however, significant differences between these groups were only pervasive across the ulna’s length with regards to CSA. Scansorial and fossorial mustelids also tended to differ from one another for ulnar traits; however, with the exception of CSA, significant differences between these groups did not extend across the length of the ulna.

**Fig. 5:**
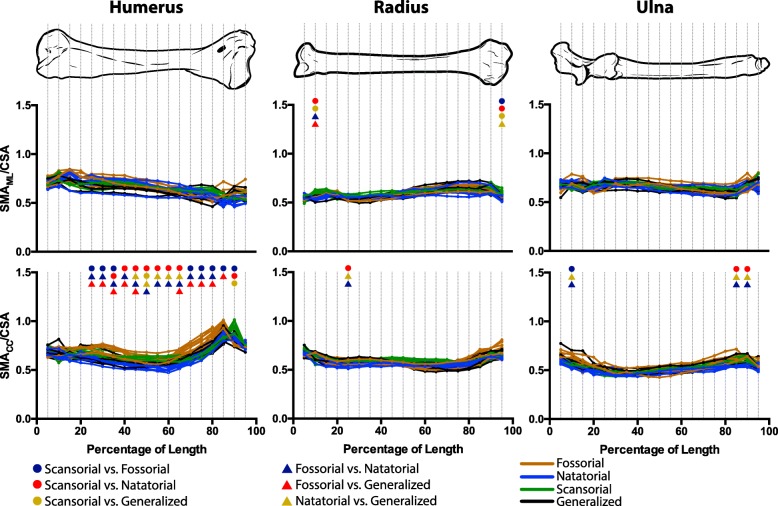
Differences in resistance ratio R for the humerus, radius, and ulna among mustelid locomotor habits. Dimensionless values of CSA and SMA were used to calculate R. Colored symbols indicate a significant difference (Adjusted *P* < 0.05) for the indicated pairwise comparison at a given increment. A lack of significant pairwise differences for a given increment indicates an overall ANOVA result of *P* > 0.0026 for that increment (the Bonferroni-corrected *P*-value). Ratios were analysed at 5% increments of bone length

Testing for significant differences among the ratio R calculated from dimensionless SMA and CSA revealed that only humeral R_CC_ showed widespread differences among differing mustelid locomotor habits (Fig. 5). These differences consisted largely of fossorial mustelids being distinct from other mustelids towards more proximal (25–45% humeral length) and distal (70–85% humeral length) regions of the humerus and natatorial mustelids contrasting to other mustelids at roughly the mid-length of the humerus (45–65%). For humeral R_ML_, there were no significant differences among groups. Likewise for the ratios of the radius and ulna, there were few, if any, significant differences along their lengths, indicating that the ratio of the resistance to bending to the resistance to compression did not significantly differ among mustelid locomotor habits. R values were largely below 1.0, indicating that mustelid long bones have a greater relative resistance to axial compression than bending.

### Trait evolution

#### Phylogenetic ANOVA

Mustelid locomotor habit was significantly correlated with phylogeny (*r* = 0.912; *P* = 0.0001). Phylogenetic ANOVAs recovered did not recover any significant differences in humeral and ulnar cross-sectional properties, with the exception of humeral CSA (*P* = 0.0210) at 85% humeral length and ulnar SMA_CC_ (*P* = 0.0410) and MOD_ML_ (*P* = 0.0490) at 20 and 30%, respectively. For the radius, there were significant differences in all cross-sectional traits at 5 and 10% of radial length (*P* ≤ 0.0250). Mustelid locomotor groups also significantly differed at 25% of radius length for CSA, SMA_ML_, and MOD_ML_ (*P* < 0.0300) and at 95% of radius length for CSA, SMA_CC_, and MOD_CC_ (*P* < 0.0500).

#### Trait evolution

**Fig. 6:**
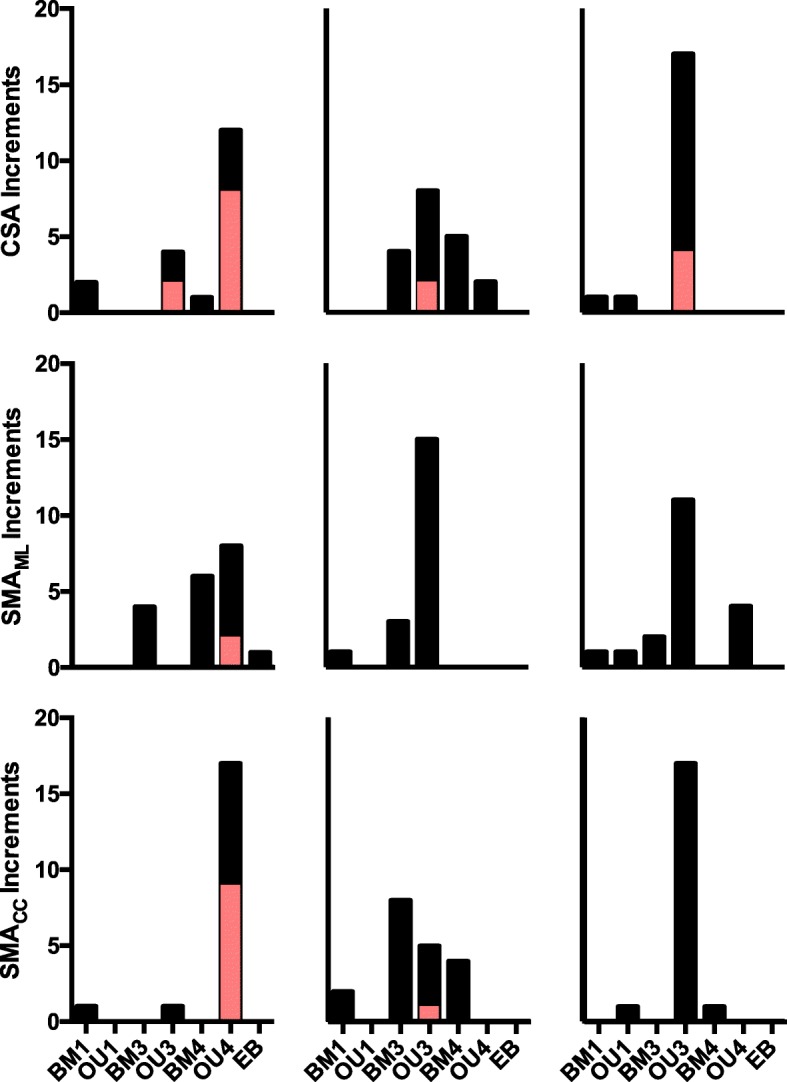
Best-fitting models of trait evolution for humeral, radial, and ulnar cross-sectional traits. For each model, the total number of increments best fit by the model are presented as black bars. In the case of the Ornstein-Uhlenbeck models, OU3 and OU4, the superimposed red bars indicate the number of OU models that also had a significant value of α, representing the strength of selection

The results of model fitting indicated that forelimb cross-sectional traits most likely evolved under either a multi-rate Brownian motion or a multi-optima OU model with distinct rates or optima for at least natatorial and climbing mustelids (Fig. 6; Additional file 2: Tables S1-S3). With regards to humeral CSA, of the 19 increments for which traits were sampled, the most prevalent model of best fit was a four-optima OU model (OU4), as the best fitting model for 12 increments (Additional file 2: Table S1). The model with next highest number of increments was the three-optima OU model (OU3) with four increments, followed by the single and four-rate Brownian motion models (BM1 and BM4, respectively) with two and one increments, respectively. With regards to humeral SMA_ML_, OU4 and BM4 were the best fitting models for eight and six increments, respectively, followed by a three-rate Brownian, which best fit four increments. For humeral SMA_CC_, the best-fitting model was overwhelmingly OU4. In the case of radial CSA, the best-fitting model was a mix largely of BM3, BM4, and OU3 (four, five, and eight increments, respectively) (Additional file 2: Table S2), whereas for radial SMA_ML_, the best-fitting models was largely OU3. In the case of radial SMA_CC_. the best-fitting model was a mix consisting primarily of BM3, OU3, and BM4 (eight, five, and four increments, respectively). Ulnar CSA and SMA_CC_ were best fit by OU3 extensively across the length of the bone (Additional file 2: Table S3). OU3 was the best-fitting model for 11 increments of ulnar SMA_ML_, followed by OU4, which was the best fit for four increments.

In the cases where an OU process was the best-fitting model, inspection of α-values revealed many instances where α could not be distinguished from a value of 0.0 (Fig. 6, red vs. black superimposed bars; Additional file 3: Tables S4-S6). In these instances, the fitted OU model did not substantially differ from a BM model of trait evolution. Only for humeral CSA and SMA_CC_, did a reasonably large fraction of best-fitting OU models possess significant α-values (Additional file 3: Table S4). For radial and ulnar CSA and SMA_ML_, only a couple of α-values proved significant, whereas for radial and ulnar SMA_ML_ and ulnar SMA_CC_ none of the α-values for best-fitting OU models were significant (Fig. 6; Additional file 3: Tables S5 and S6). Additionally, the confidence limits for α with regards to traits for all three bones – most markedly those of those of the ulna – suffered from implausibly wide confidence limits, indicating high difficulty in fitting OU models to ulnar CSA, SMA_ML_, and SMA_CC_.

## Discussion

### Cross-sectional morphology and biomechanical advantage

Our results demonstrate that the cross-sectional morphology of long bones can differ among specialized locomotor habits in Mustelidae, a functionally diverse and speciose lineage within Carnivora. These findings fit well with broader patterns throughout the vertebrate skeleton, indicating linkages of form, function and behavior or performance; in other words, that bone geometry grossly reflects loading patterns. For example, differences in long bone cross-sectional traits have been reported in birds of differing locomotor modes [34, 92] and primates differing in slow climbing, suspensory, and leaping locomotor habits [16, 44, 86]. While many prior studies have focused on external bone dimensions and their relationships with higher-level biological factors such as locomotion, we have contributed a new, focused analysis of Mustelidae using the valuable perspective that analysis of internal (i.e. cross-sectional) bone dimensions can bring. Our findings have some general correspondences with similar analyses by Doube et al. [18–20]; cf. shapes of curves in our Figs. 4 and 5) and others, but the Mustelidae-specific insights are important and novel.

In line with our prediction, natatorial and fossorial mustelids tended to have greater values of cross-sectional traits than remaining mustelids (Fig. 4 and Additional file 1: Figure S1), with natatorial and scansorial mustelids possessing respectively the highest and lowest values of cross-sectional traits. Natatorial mustelids significantly differed from scansorial (red circles) and generalized mustelids (bronze triangles) in all cross-sectional traits of the humerus and radius, with these differences being rather extensive along these bones’ lengths. Natatorial mustelids also significantly differed from scansorial and generalized mustelids in ulnar cross-sectional traits, though to a much lesser extent, apart from ulnar CSA. In contrast, fossorial mustelids tended to significantly differ primarily from scansorial mustelids (navy circles) in humeral and radial cross-sectional traits; however, differences in ulnar SMA and MOD were not prevalent across the entire length of the ulna. We found that significant differences between fossorial and generalized mustelids (red triangles) only occurred in humeral SMA and MOD at localized regions along this bone’s length. Thus in mustelids, any possible locomotion-distinct phenotypes associated with cross-sectional morphology do not necessarily encompass all forelimb bones or all cross-sectional traits. Moreover, the four locomotor habits within Mustelidae sampled here likely are not each characterized by a distinct cross-sectional morphology, because generalized mustelids only rarely differed in cross-sectional traits from both scansorial and fossorial species (bronze circles and red triangles, respectively).

The low values of cross-sectional traits of the forelimb skeleton in scansorial mustelids correspond to the greater gracility of their forelimb skeleton [27, 53] and the relatively elongate and lightweight limbs of scansorial mammals in general [51, 52]. The gracile and elongate forelimb skeleton of martens, though not as extreme as in other scansorial carnivorans [64], likely confers advantages in bridging discontinuities in supports (e.g., tree branches) while climbing [13]. Moreover, Cartmill [13] argued that larger body sizes may hamper climbing ability; therefore it is also seems plausible that overly robust or more massive limbs may also be disadvantageous for a climbing lifestyle.

Greater values of cross-sectional traits strongly distinguishing natatorial mustelids from scansorial, generalized, and, to a lesser extent, fossorial mustelids (Fig. 4 and Additional file 1: Figure S1: red circles, bronze and navy triangles) indicate that otters have humeri, radii, and, to a lesser degree, ulnae with greater relative resistance to compression (i.e., CSA) and bending (i.e., SMA) and greater structural strength (i.e., MOD) than mustelids of other locomotor habits. The greater values of cross-sectional traits for natatorial mustelids would be advantageous for swimming by drag-based propulsion, though the degree to which forelimbs function in swimming varies among otter species. Notably, the forelimbs of sea otters (*Enhydra lutris*) do not play a role in swimming but are extensively involved in tool use and prey manipulation, such as hammering open or prying prey loose [50, 68]. It could be that the forces generated in this behavior could require a forelimb skeleton structurally stronger than other mustelids; however, there currently appears to be no published data on the mechanics of tool use in this species.

However, greater values of cross-sectional traits – and consequently the increased load resistance they offer – are likely not critical to swimming in mustelids. Recent work comparing bone loading in turtles, both during walking and swimming, found significantly lower bone strains during swimming than walking, likely due to buoyant forces removing the need for the limbs to support body weight despite their roles in providing thrust during locomotion [103, 104].

An alternative and more likely, though not mutually exclusive, explanation would be the need for thicker bones to help counteract buoyancy during subsurface swimming [42, 43]. Given that natatorial mustelids have the highest values of cross-sectional traits, it strongly suggests that the need to counteract buoyancy may have a stronger influence upon cross-sectional morphology than any increased resistance to the musculoskeletal loads imposed by specialized limb functions occurring in mustelids. An exception to the general trend among otters is the small-clawed otter (*Amblonyx cinereus*), which lies comfortably in the range of scansorial mustelids. Notably, this species possesses rather gracile long bones more comparable to scansorial mustelids [27, 53] than to other otters, with its humerus further lacking the strong anterior bowing characteristic of other otters ([9]; pers. obs.). Moreover, this species forages somewhat more terrestrially where it occurs sympatrically with Eurasian otters (*Lutra lutra*) and smooth-coated otters (*Lutrogale perspicillata*) [55], and the webbing is incomplete between its digits [60], so it could be considered less aquatic than other otter species.

Fossorial mustelids have high values of cross-sectional traits compared to scansorial and generalized mustelids (Fig. 4 and Additional file 1: Figure S1: brown curves), likely due to the limbs having to function in soil, which has a high density. Although the degree of fossoriality may vary among taxa [84], most badgers and other fossorial mustelids (e.g., zorilla, *Ictonyx striatus*) dig as a means of foraging and may dig their own burrows [56, 59, 73, 77, 93, 98]. However, some badgers display exceptional digging ability, including rapid digging [56], digging extensive burrow systems [83], digging a new den every night [65], and burying food items several times larger than themselves as a cache [31]. Interestingly, significant differences in SMA and MOD between fossorial and other mustelids were noticeably not as widespread in the ulna as in the humerus and radius. This is surprising given the insertion of the triceps muscle group, which is highly specialized with an angular head in mustelids [25], onto the olecranon process, and the triceps’ highly integral role in exerting force during scratch digging [39, 40, 71]. However, these distinct results for the ulna may be due to the trochlea of the humerus and the trochlear notch of the ulna restricting its movement to flexion and extension relative to the humerus regardless of specializations in limb function. Thus, the ulna cannot exhibit long axis rotation unlike the radius, and thus may experience a lower diversity of loading regimes than the latter bone. Moreover, given that the distal articulation of the radius has much broader contact with the carpus than that of the ulna, it could be possible that the radius receives more of the mechanical loads transmitted proximally along the forelimb by the manus than the ulna, and, if so, this may be reflected in the differences in radial cross-sectional morphology among mustelid locomotor habits. This discrepancy in results among the humerus, radius, and ulna suggests that the loading of limb bones during digging may be complex, with differing bones operating at different loads and safety factors (e.g. perhaps fitting the “mixed chain” hypothesis; [4]).

In addition to function, size may be another factor influencing forelimb morphology in mustelids. In particular, greater values of cross-sectional traits are generally associated with larger body sizes in many mammals [18, 19] and birds [20]. Plotting dimensionless values of mustelid cross-sectional traits against body mass reveals a complicated relationship with body size (Additional file 4: Figure S2). Otters, which include the most massive mustelids, appear to have an allometric trajectory distinct from other mustelids’. However, for a given body mass where multiple locomotor habits coincide, scansorial mustelids have smaller values of cross-sectional traits than either fossorial and generalist mustelids do. This differentiation of locomotor habits for a given body mass suggests that our results are not solely due to the influence of size (i.e. scaling). Rather our results appear subject to the mixed influences of locomotor habit and size.

### Resistance to bending vs. compression

The ratio R revealed that, by and large, differences in locomotor habit are not associated with a trade-off in resistance to bending vs. compression (Fig. 5). Humeral R_CC_ was an exception to this, with significant differences occurring among mustelids locomotor habits between 25 and 90% of humeral length. Notably, in contrast to our separate tests of individual cross-sectional traits, R_CC_ distinguished fossorial and natatorial mustelids, with badgers having significantly greater values of R_CC_ than otters. This result suggests that, in the case of badger humeri, possible selection with regards to fossoriality in mustelids may pertain more to the ratio of resistance to particular loading regimes than the absolute resistance to a single loading regime. Compared to otters, badgers exhibit humeri with relatively greater resistance to bending about the cranio-caudal axis (Fig. 1) relative to the total amount of bone tissue comprising their humeral cross-section. In other words, badgers have a wider distribution of bone tissue in their humeral cross-section than otters in spite of having a lower amount of overall bone tissue within their cross-section. This result concurs with our earlier finding of badgers having more robust forelimb long bones (in terms of external dimensions; [53]) and our current finding that badgers have lower values of CSA than otters.

Humeral R_CC_ also distinguished natatorial mustelids from scansorial mustelids, with martens having greater SMA_CC_ relative to CSA, and fossorial mustelids from generalized mustelids, further suggesting that the humerus’ relative resistance to different loading regimes may distinguish mustelid locomotor habits. The low values of humeral R_CC_ displayed by natatorial mustelids likely reflect the medio-laterally compressed humeral diaphysis of otters, with such compression being common for aquatic tetrapods [104]. These differences in R_CC_ suggest there may be differences in incurred loading regime as forelimbs conduct different functions in mustelids. While this is an exciting topic of investigation, it unfortunately is beyond the scope of our study.

Apart from humeral R_CC_, there is striking uniformity among other ratios of R (Fig. 5) in Mustelidae. This uniformity suggests that the relative resistance to different loading regimes is not fundamental to functional specializations of the limb and that a single ‘design’ of relative loading resistance allows for disparate limb functions. Furthermore, the uniformity in R values suggests that distribution of bone tissue (i.e., SMA) relative to the total amount of bone tissue (i.e., CSA) of a cross-section may possibly be phylogenetically conserved, or biomechanically or developmentally constrained, at least for the radius and ulna. A conserved internal morphology of the ulna is particularly surprising when considering mammals more broadly, given the variability of the ulna’s external dimensions in terms of its reduction, relative olecranon length, and robustness in relation to specialized limb functions [87, 88], though admittedly mustelids in and of themselves do not display such wide extremes in ulnar morphology. It remains unclear if our findings would, however, relate to the mesopodium or autopodium (carpus/manus).

### Evolution of cross-sectional morphology

Within Mustelidae, locomotor habit is intimately linked with phylogeny. Notably, natatorial species evolved from a single ancestor within our sampled mustelids, as is the case for scansorial species (Fig. 1). Among our sample, there is one instance of convergence in fossorial limb function (*Ictonyx striatus*), though until recently there was thought to be more convergence in fossoriality in Mustelidae [89]. The preponderance of non-significant results for phylogenetic ANOVAs further underscores that phylogeny is a strong component of the observed morphological variation in Mustelidae. However, our lack of significant findings with phylogenetic ANOVAs goes against the known biomechanical relevance of cross-sectional bone dimensions for many mustelid species, particularly otters (e.g., [42]). While phylogenetic ANOVAs are vital to address the influence of shared ancestry upon trait variation, such analyses by themselves could lead to faulty interpretations of how morphology relates to biomechanical function. In turn, while standard ANOVAs are able to discern morphological differences relevant to biomechanics, they obviously fail to address the role of phylogeny in trait variation. Thus, the pairing of both ahistorical and historical analyses is required for a more comprehensive view of the evolution of biomechanical traits.

Fitting models of trait evolution uncovered that the most likely pattern of evolution with regards to the cross-sectional traits of the humerus, radius, and ulna was either a multi-rate Brownian motion model (BM3/BM4) or a multi-optima Ornstein-Uhlenbeck model (OU3/OU4) (Fig. 6). These models distinguish either distinct rates of evolution (Brownian motion models) or evolution towards distinct phenotypic optima (Ornstein-Uhlenbeck models) for the differing locomotor habits within Mustelidae. Both of these models propose that natatorial and scansorial mustelids morphologically diverged from one another and remaining mustelids, either by evolving under differing rates of Brownian motion or towards distinct adaptive optima. Moreover, finding BM4 and OU4 as the best-fitting model indicates that each locomotor habit within Mustelidae is tied to a divergence in forelimb cross-sectional traits. This result is in line with these two locomotor habits being the extremes of cross-sectional morphology in mustelid long bones.

The prevalence of OU models as the best-fitting models would suggest that the locomotor diversity among mustelids is the result of evolution towards distinct phenotypes ‘optimal’ for the biomechanical demands of a given locomotor habit. However, inspection of α, commonly interpreted as the strength of selection in OU models [35], is crucial prior to accepting an OU model as the most plausible mode of evolution for a given trait [15]. When α does not significantly differ from 0.0, then the OU model is equivalent to a Brownian motion model [11]. Inspection of α-values in instances where OU models were the best-fitting models revealed numerous instances where α could not be distinguished from 0.0 (Fig. 6 and Additional file 3: Tables S4-S6). The outperformance by the OU model vs. the BM model in these instances was due to the additional parameters of the OU model affording the best description of the data’s variance outside of the model’s biological relevance [15].

It thus appears that the cross-sectional morphology of the mustelid humerus, radius, and ulna has evolved predominantly due to a multi-rate Brownian motion process. Under such a mode of evolution, the distinct cross-sectional morphologies of mustelid locomotor habits are associated with a distinct rate of phenotypic evolution, and it is possible that these differences in rate are associated with different constraints upon the evolution of cross-sectional morphology in mustelids (see [74, 96]). Such constraints regarding mustelid limbs could be the biomechanical benefits of thinner, and presumably more lightweight, bones associated with a climbing lifestyle or more robust bones associated with an aquatic lifestyle (see above). Alternatively, cross-sectional morphology may have been under selection at one point during mustelid evolution, with resulting changes in morphology being conserved among later divergences of mustelids (i.e., phylogenetic inertia in the trait). This would be in contrast to a continuous selective regime acting across the branches associated with those later divergences (as is the case in an OU model).

This overall result contrasts with the likely mode of evolution for the external dimensions of the forelimb skeleton (e.g., lengths, diameters, and muscle in-lever lengths). The external dimensions of the forelimb skeleton likely evolved adaptively, with adaptive peaks distinguishing scansorial from remaining mustelids in terms of the length of muscle in-levers and long bone gracility [53]. Then again, the contrasting results of the current study and those of Kilbourne [53] might be due to sample size. In the current study, we restricted our sample size to seven taxa per locomotor habit for a total of 28 taxa, whereas Kilbourne [53] sampled as many mustelid species as possible for a total of 41 taxa. However, another possible explanation may be that the different, functionally relevant traits within a single functioning organ may evolve by different processes in mustelids. These results also raise the question of how do differing traits, with different biomechanical functions (e.g., the mechanical advantage offered by muscle in-levers vs. the bending resistance offered by SMA), contribute to the overall adaptations occurring in a limb? This question merits future focus in trait evolution studies combining different kinds of traits, though current methods may be ill equipped to address it [1].

## Conclusions

The cross-sectional morphologies of the humerus, radius, and ulna exhibit differences among mustelid locomotor specializations. In particular, scansorial and natatorial mustelids are strongly associated with more gracile and more robust cross-sections, respectively. Comparing the ratio of bending to compression resistance in mustelids finds a largely uniform ratio across locomotor habits, excluding humeral R_CC_, suggesting a possible constraint upon forelimb morphology in mustelids (e.g. conserved distribution of bone tissue relative to the anatomical axes for a given CSA). However, the phylogenetic relationships among mustelids have had considerable influence upon the disparity of mustelid cross-sectional traits. Cross-sectional traits appear to most likely have evolved according to a multi-rate Brownian motion process, with distinct rates of Brownian motion evolution for scansorial and natatorial mustelids. This is in contrast to previous findings when fitting trait diversification models to the external traits (e.g., lengths, diameters) of the forelimb skeleton, which appear to have diversified adaptively. This difference in results may be due to differences in sample size or the possibility that biomechanical traits with differing roles undergo substantially different modes of evolution within a given organ.

## Additional files

Additional file 1: **Figure S1.** Differences in humeral radial, and ulnar section modulus among mustelid locomotor habits. Differences in MOD_ML_ and MOD_CC_ were tested at 5% increments along each bone’s length. MOD was rendered dimensionless by respectively taking the third root and dividing by bone length. Colored symbols indicate a significant difference (Adjusted *P* < 0.05) for the indicated pairwise comparison at a given increment. A lack of significant pairwise differences for a given increment indicates an overall ANOVA result of *P* > 0.0026 for that increment (the Bonferroni-corrected *P*-value). (EPS 7340 kb)

Additional file 2: **Table S1.** Akaike weights for trait diversification models fitted to humeral cross-sectional traits. **Table S2.** Akaike weights for trait diversification models fitted to radial cross-sectional traits. **Table S3.** Akaike weights for trait diversification models fitted to ulnar cross-sectional traits. (DOCX 58 kb)

Additional file 3: **Table S4.** Significance and magnitude of alpha values for OU models that were determined to be the best fitting model for humeral cross-sectional traits. **Table S5.** Significance and magnitude of alpha values for OU models that were determined to be the best fitting model for radial cross-sectional traits. **Table S6.** Significance and magnitude of alpha values for OU models that were determined to be the best fitting model for ulnar cross-sectional traits. (DOCX 51 kb)

Additional file 4: **Figure S2.** Allometric patterns present within mustelid humeral cross-sectional traits, here represented by dimensionless CSA and SMA plotted against body mass on a logarithmic scale. The rows of blue, red and grey numbers on each plot show the scaling exponents (and 95% confidence intervals) for each regression (for behavioral sub-groups or all of Mustelidae). All regressions shown have exponents > 0, and hence slight positive allometry is evident for all parameters quantified. Regressions were performed with the R package smatr [99]. (EPS 3570 kb)
