# Supplementary material for: Morphological diversification of biomechanical traits: mustelid locomotor specializations and the macroevolution of long bone cross-sectional morphology
Source: BMC Evol Biol. 2019 Jan 30;19:37. doi: 10.1186/s12862-019-1349-8 (PMC6354431; doi:10.1186/s12862-019-1349-8)
Supplement: Supplementary file 2 — Table S1. Akaike weights for trait diversification models fitted to humeral cross-sectional traits. Table S2. Akaike weights for trait diversification models fitted to radial cross-sectional traits. Table S3. Akaike weights for trait diversification models fitted to ulnar cross-sectional traits. (DOCX 58 kb) [file 12862_2019_1349_MOESM2_ESM.docx]

**Table S1**. Akaike weights for trait diversification models fitted to humeral cross-sectional traits. Models fitted include Brownian motion models with one, three, and four rates (BM1, BM3, and B4, respectively) and Ornstein-Uhlenbeck models with one, three, and four phenotypic optima (OU1, OU3, and OU4, respectively). BM3 and OU3 have their respective distinct rates and optima based upon three functional categories: scansorial, natatorial, and a third category combining fossorial and generalized mustelids. BM4 and OU4 have their respective rates and optima based upon each of the four locomotor categories occurring within extant mustelids: fossorial, generalized, natatorial, and scansorial.

|  | **Humeral CSA** | | | | | | | | | | | | | | | | | | |
| --- | --- | --- | --- | --- | --- | --- | --- | --- | --- | --- | --- | --- | --- | --- | --- | --- | --- | --- | --- |
| **Model** | **5** | **10** | **15** | **20** | **25** | **30** | **35** | **40** | **45** | **50** | **55** | **60** | **65** | **70** | **75** | **80** | **85** | **90** | **95** |
| BM1 | 18.8 | 0.0 | 17.5 | 5.6 | 1.3 | 0.7 | 0.6 | 0.4 | 0.2 | 0.1 | 0.3 | 0.7 | 1.2 | 0.5 | 0.2 | 0.2 | **36.0** | 0.2 | **41.6** |
| OU1 | 5.4 | 0.0 | 5.1 | 1.6 | 0.4 | 0.2 | 0.2 | 0.1 | 0.1 | 0.0 | 0.1 | 0.2 | 0.4 | 0.2 | 0.1 | 0.1 | 10.2 | 0.3 | 12.7 |
| BM3 | 1.5 | 0.0 | 6.0 | 2.9 | 1.3 | 7.8 | 0.6 | 0.9 | 0.4 | 0.1 | 0.5 | 3.7 | 8.8 | 0.6 | 0.1 | 0.1 | 14.3 | 0.0 | 3.2 |
| OU3 | **44.8** | 38.9 | 12.4 | 21.8 | 43.3 | 33.7 | **48.9** | **48.4** | 40.8 | **48.5** | 30.6 | 16.3 | 11.8 | 19.3 | 12.0 | 13.2 | 10.9 | 17.8 | 24.5 |
| BM4 | 0.4 | 0.0 | 1.4 | 0.6 | 4.3 | **36.4** | 8.7 | 4.4 | 8.1 | 2.9 | 5.9 | 21.8 | 27.1 | 5.3 | 0.8 | 0.1 | 3.9 | 0.0 | 1.1 |
| OU4 | 23.7 | **61.0** | **52.6** | **66.0** | **49.0** | 20.9 | 41.8 | 45.6 | **50.4** | 48.3 | **62.5** | **57.1** | **50.4** | **74.1** | **86.8** | **86.4** | 14.1 | **81.4** | 4.2 |
| EB | 5.4 | 0.0 | 5.1 | 1.6 | 0.4 | 0.2 | 0.2 | 0.1 | 0.1 | 0.0 | 0.1 | 0.2 | 0.4 | 0.2 | 0.1 | 0.1 | 10.7 | 0.3 | 12.7 |
|  | **Humeral SMA_ML_** | | | | | | | | | | | | | | | | | | |
| BM1 | 22.9 | 0.3 | 9.2 | 4.9 | 0.6 | 0.3 | 0.3 | 0.1 | 0.1 | 0.1 | 0.2 | 0.2 | 0.1 | 0.3 | 0.2 | 2.8 | 17.6 | 1.6 | 22.9 |
| OU1 | 7.2 | 0.2 | 3.1 | 1.4 | 0.2 | 0.2 | 0.2 | 0.1 | 0.1 | 0.1 | 0.1 | 0.1 | 0.0 | 0.1 | 01 | 0.8 | 5.0 | 2.9 | 7.2 |
| BM3 | 7.0 | 0.0 | 12.7 | **41.2** | **62.2** | **52.1** | 10.5 | 10.2 | 4.7 | 1.8 | 6.6 | 29.4 | **54.8** | 22.7 | 2.1 | 13.0 | 16.1 | 0.2 | 7.0 |
| OU3 | 15.9 | 14.5 | 5.5 | 2.4 | 2.1 | 3.3 | 6.3 | 3.0 | 5.6 | 12.0 | 9.3 | 2.3 | 1.2 | 25.2 | 41.3 | 12.5 | 1.2 | 15.5 | 15.9 |
| BM4 | 2.3 | 0.0 | 3.1 | 16.5 | 14.9 | 30.9 | **65.2** | **78.4** | **74.5** | **60.9** | **57.3** | **61.0** | 41.7 | 21.6 | 7.4 | 6.3 | 4.8 | 0.4 | 2.3 |
| OU4 | **37.5** | **84.7** | **63.3** | 32.3 | 19.7 | 13.0 | 17.4 | 8.0 | 14.8 | 25.1 | 26.4 | 6.9 | 2.1 | **30.1** | **48.9** | **63.8** | 0.8 | **76.5** | **37.5** |
| EB | 7.2 | 0.2 | 3.1 | 1.4 | 0.2 | 0.2 | 0.2 | 0.1 | 0.1 | 0.1 | 0.1 | 0.1 | 0.0 | 0.1 | 0.1 | 0.8 | **54.5** | 2.9 | 7.2 |
|  | **Humeral SMA_CC_** | | | | | | | | | | | | | | | | | | |
| BM1 | 3.7 | 0.2 | 30.1 | 15.9 | 12.1 | 16.9 | 16.4 | 10.2 | 5.0 | 3.8 | 3.4 | 7.8 | 16.7 | 4.0 | 1.5 | 1.7 | 6.9 | 1.8 | **44.8** |
| OU1 | 1.1 | 0.1 | 8.6 | 5.9 | 5.0 | 6.1 | 5.7 | 3.5 | 1.9 | 1.5 | 1.3 | 2.5 | 4.8 | 1.5 | 0.6 | 0.6 | 2.1 | 1.0 | 12.8 |
| BM3 | 2.2 | 0.0 | 8.1 | 5.5 | 3.0 | 4.0 | 4.6 | 5.8 | 4.0 | 2.4 | 2.2 | 3.9 | 10.4 | 1.6 | 0.3 | 0.3 | 2.1 | 0.2 | 11.3 |
| OU3 | **55.0** | 44.3 | 10.3 | 10.8 | 9.6 | 10.6 | 8.9 | 7.3 | 8.0 | 13.2 | 13.1 | 7.5 | 7.3 | 5.3 | 3.3 | 3.3 | 6.6 | 14.7 | 12.7 |
| BM4 | 2.0 | 0.2 | 2.2 | 1.7 | 1.8 | 1.7 | 2.2 | 4.0 | 3.1 | 1.6 | 1.1 | 1.1 | 2.7 | 0.9 | 0.4 | 0.2 | 2.2 | 2.7 | 2.89 |
| OU4 | 34.9 | **55.0** | **32.0** | **54.4** | **63.4** | **54.5** | **56.5** | **65.8** | **76.2** | **75.9** | **77.6** | **74.8** | **53.3** | **85.2** | **93.2** | **93.4** | **77.9** | **78.5** | 2.8 |
| EB | 1.1 | 0.1 | 8.6 | 5.9 | 5.0 | 6.1 | 5.7 | 3.5 | 1.9 | 1.5 | 1.3 | 2.5 | 4.8 | 1.5 | 0.6 | 0.6 | 2.1 | 1.0 | 12.8 |

**Table S2**. Akaike weights for trait diversification models fitted to radial cross-sectional traits. Models fitted include Brownian motion models with one, three, and four rates (BM1, BM3, and B4, respectively) and Ornstein-Uhlenbeck models with one, three, and four phenotypic optima (OU1, OU3, and OU4, respectively). BM3 and OU3 have their respective distinct rates and optima based upon three functional categories: scansorial, natatorial, and a third category combining fossorial and generalized mustelids. BM4 and OU4 have their respective rates and optima based

|  | **Radial CSA** | | | | | | | | | | | | | | | | | | |
| --- | --- | --- | --- | --- | --- | --- | --- | --- | --- | --- | --- | --- | --- | --- | --- | --- | --- | --- | --- |
| **Model** | **5** | **10** | **15** | **20** | **25** | **30** | **35** | **40** | **45** | **50** | **55** | **60** | **65** | **70** | **75** | **80** | **85** | **90** | **95** |
| BM1 | 9.6 | 0.6 | 0.7 | 0.0 | 0.1 | 0.1 | 0.1 | 0.1 | 0.2 | 0.1 | 0.1 | 0.1 | 0.1 | 0.0 | 0.0 | 0.2 | 4.3 | 0.4 | 25.4 |
| OU1 | 3.4 | 0.3 | 0.3 | 0.0 | 0.0 | 0.0 | 0.0 | 0.1 | 0.1 | 0.0 | 0.0 | 0.0 | 0.0 | 0.0 | 0.0 | 0.0 | 1.2 | 0.3 | 7.3 |
| BM3 | 2.5 | **46.6** | 28.9 | **49.3** | 13.9 | 0.9 | 1.0 | 1.2 | 10.5 | 8.3 | 2.8 | 2.9 | 7.4 | 38.4 | **67.2** | **70.1** | 14.5 | 0.3 | 3.9 |
| OU3 | 36.4 | 18.2 | **40.7** | 15.3 | **58.9** | **77.5** | **76.9** | **76.0** | **53.0** | 31.9 | 23.2 | 38.5 | 19.1 | 10.2 | 2.5 | 0.6 | **50.6** | **69.2** | 4.4 |
| BM4 | 0.8 | 26.4 | 15.5 | 30.3 | 12.2 | 0.9 | 1.6 | 3.5 | 21.3 | **50.4** | **66.9** | **46.7** | **67.3** | **48.1** | 29.2 | 28.8 | 3.1 | 0.1 | 2.8 |
| OU4 | **43.8** | 7.7 | 13.7 | 4.91 | 14.9 | 20.5 | 20.3 | 19.0 | 14.8 | 9.2 | 6.8 | 11.7 | 6.0 | 3.2 | 0.9 | 0.2 | 25.0 | 29.4 | **48.9** |
| EB | 3.4 | 0.3 | 0.3 | 0.0 | 0.0 | 0.0 | 0.0 | 0.1 | 0.1 | 0.0 | 0.0 | 0.0 | 0.0 | 0.0 | 0.0 | 0.0 | 1.2 | 0.3 | 7.3 |
|  | **Radial SMA_ML_** | | | | | | | | | | | | | | | | | | |
| BM1 | 6.1 | 7.0 | **1.2** | 0.0 | 0.0 | 0.1 | 0.1 | 0.3 | 0.3 | 0.2 | 0.2 | 0.2 | 0.2 | 0.6 | 2.6 | 11.6 | 13.7 | 0.4 | **48.0** |
| OU1 | 2.0 | 4.3 | 1.6 | 0.0 | 0.0 | 0.1 | 0.1 | 0.2 | 0.3 | 0.2 | 0.3 | 0.3 | 0.2 | 0.4 | 1.5 | 4.6 | 5.8 | 0.6 | 15.4 |
| BM3 | 2.8 | **56.1** | 6.1 | **81.2** | **56.8** | 1.7 | 0.7 | 1.1 | 1.7 | 0.1 | 0.0 | 0.0 | 0.1 | 1.0 | 4.6 | 20.9 | 12.5 | 0.2 | 3.6 |
| OU3 | **43.0** | 6.5 | **64.3** | 3.7 | 26.2 | **77.7** | **78.3** | **76.1** | **73.3** | **69.7** | **67.9** | **67.6** | **69.3** | **57.5** | **55.8** | **26.9** | **42.6** | **75.9** | 2.5 |
| BM4 | 2.3 | 18.3 | 1.4 | 13.6 | 10.6 | 0.5 | 0.3 | 0.5 | 1.8 | 1.1 | 1.5 | 1.7 | 4.8 | 20.3 | 14.7 | 23.4 | 5.9 | 0.3 | 1.9 |
| OU4 | 41.9 | 3.6 | 23.7 | 1.3 | 6.4 | 19.8 | 20.5 | 21.5 | 22.4 | 28.5 | 29.7 | 29.8 | 25.1 | 19.8 | 19.4 | 7.9 | 13.8 | 21.9 | 13.2 |
| EB | 2.0 | 4.3 | 1.6 | 0.0 | 0.0 | 0.1 | 0.1 | 0.2 | 0.3 | 0.2 | 0.3 | 0.3 | 0.2 | 0.4 | 1.5 | 4.6 | 5.8 | 0.6 | 15.4 |
|  | **Radial SMA_CC_** | | | | | | | | | | | | | | | | | | |
| BM1 | 3.3 | 1.1 | 3.2 | 1.8 | 1.5 | 0.7 | 0.4 | 0.1 | 0.0 | 0.0 | 0.0 | 0.0 | 0.0 | 0.0 | 0.1 | 1.2 | **36.7** | 9.0 | **25.7** |
| OU1 | 1.4 | 0.6 | 1.8 | 1.1 | 1.1 | 0.6 | 0.3 | 0.1 | 0.0 | 0.0 | 0.0 | 0.0 | 0.0 | 0.0 | 0.0 | 0.3 | 10.4 | 3.8 | 9.6 |
| BM3 | 5.8 | **59.8** | **51.4** | **34.2** | 7.2 | 4.7 | 6.2 | 3.6 | 1.8 | 7.0 | 21.1 | **54.2** | **81.1** | **83.2** | **83.5** | **78.8** | 17.7 | 9.7 | 17.1 |
| OU3 | **44.0** | 5.2 | 7.1 | 32.1 | **66.2** | **68.9** | **58.6** | 31.4 | 1.6 | 0.1 | 0.04 | 0.0 | 0.1 | 0.2 | 0.5 | 1.1 | 11.9 | **35.8** | 13.8 |
| BM4 | 10.5 | 30.2 | 32.2 | 19.9 | 6.5 | 6.0 | 18.9 | **56.9** | **96.2** | **92.9** | **78.9** | 45.8 | 18.7 | 16.5 | 15.6 | 17.8 | 5.6 | 2.4 | 8.8 |
| OU4 | 33.4 | 2.6 | 2.5 | 9.9 | 16.5 | 18.6 | 15.2 | 7.7 | 0.4 | 0.0 | 0.0 | 0.0 | 0.0 | 0.1 | 0.3 | 0.4 | 6.4 | 35.6 | 15.6 |
| EB | 1.4 | 0.6 | 1.8 | 1.1 | 1.1 | 0.6 | 0.3 | 0.1 | 0.0 | 0.0 | 0.0 | 0.0 | 0.0 | 0.0 | 0.0 | 0.4 | 11.1 | 3.8 | 9.6 |

upon each of the four locomotor categories occurring within extant mustelids: fossorial, generalized, natatorial, and scansorial.

**Table S3**. Akaike weights for trait diversification models fitted to ulnar cross-sectional traits. Models fitted include Brownian motion models with one, three, and four rates (BM1, BM3, and B4, respectively) and Ornstein-Uhlenbeck models with one, three, and four phenotypic optima (OU1, OU3, and OU4, respectively). BM3 and OU3 have their respective distinct rates and optima based upon three functional categories: scansorial, natatorial, and a third category combining fossorial and generalized mustelids. BM4 and OU4 have their respective rates and optima based

|  | **Ulnar CSA** | | | | | | | | | | | | | | | | | | |
| --- | --- | --- | --- | --- | --- | --- | --- | --- | --- | --- | --- | --- | --- | --- | --- | --- | --- | --- | --- |
| **Model** | **5** | **10** | **15** | **20** | **25** | **30** | **35** | **40** | **45** | **50** | **55** | **60** | **65** | **70** | **75** | **80** | **85** | **90** | **95** |
| BM1 | 0.8 | 3.8 | 0.01 | **30.8** | 0.6 | 3.4 | 6.7 | 2.3 | 2.0 | 1.0 | 1.1 | 1.5 | 0.6 | 0.5 | 0.1 | 0.1 | 0.3 | 0.1 | 14.7 |
| OU1 | 1.3 | 6.7 | 0.2 | 21.1 | 1.6 | 2.1 | 2.9 | 1.6 | 1.9 | 0.9 | 0.5 | 0.6 | 0.3 | 0.2 | 0.1 | 0.1 | 0.1 | 0.1 | **25.3** |
| BM3 | 4.3 | 3.2 | 0.6 | 2.4 | 0.0 | 9.4 | 7.7 | 1.9 | 1.3 | 1.5 | 4.5 | 21.5 | 3.1 | 8.7 | 2.2 | 0.6 | 11.4 | 0.1 | 5.1 |
| OU3 | **51.8** | **60.9** | **79.4** | 12.9 | **59.8** | **56.5** | **53.6** | **66.3** | **66.9** | **71.4** | **70.5** | **54.8** | **75.4** | **73.6** | **81.0** | **83.4** | **70.3** | **81.9** | 14.6 |
| BM4 | 9.7 | 0.7 | 0.3 | 7.6 | 0.1 | 2.1 | 2.4 | 0.5 | 0.3 | 0.4 | 0.9 | 4.2 | 0.6 | 1.8 | 0.7 | 0.2 | 2.1 | 0.0 | 10.6 |
| OU4 | 30.9 | 17.9 | 19.2 | 4.1 | 36.3 | 24.4 | 23.8 | 25.8 | 25.7 | 23.9 | 21.9 | 16.7 | 19.7 | 15.0 | 15.8 | 15.6 | 15.7 | 17.8 | 4.5 |
| EB | 1.3 | 6.7 | 0.2 | 21.1 | 1.6 | 2.1 | 2.9 | 1.6 | 1.9 | 0.9 | 0.5 | 0.6 | 0.3 | 0.2 | 0.1 | 0.1 | 0.1 | 0.1 | 25.3 |
|  | **Ulnar SMA_ML_** | | | | | | | | | | | | | | | | | | |
| BM1 | 0.5 | 0.3 | 0.4 | **39.4** | 9.9 | 6.2 | 17.9 | 14.4 | 12.0 | 6.2 | 4.5 | 3.7 | 3.2 | 4.0 | 3.5 | 1.9 | 6.5 | 1.0 | 3.4 |
| OU1 | 0.3 | 0.4 | 2.6 | 14.6 | 9.3 | 2.7 | 7.0 | 8.7 | 9.4 | 5.2 | 2.8 | 2.2 | 2.3 | 2.3 | 2.0 | 1.2 | 3.1 | 1.3 | **43.0** |
| BM3 | 2.3 | 9.6 | 8.7 | 7.2 | 1.4 | **54.1** | **23.2** | 10.3 | 8.5 | 10.1 | 15.8 | 22.0 | 9.8 | 22.7 | 4.5 | 1.2 | 4.5 | 0.9 | 1.1 |
| OU3 | **78.1** | **73.2** | **47.6** | 10.5 | **46.2** | 11.0 | 13.3 | **24.7** | 27.1 | 27.9 | 25.3 | 28.1 | **46.2** | **39.5** | **57.2** | **68.5** | **52.7** | **75.7** | 5.6 |
| BM4 | 2.5 | 2.5 | 2.6 | 8.1 | 0.3 | 14.9 | 21.1 | 16.5 | 6.2 | 5.6 | 6.7 | 9.6 | 2.4 | 4.9 | 1.1 | 0.3 | 1.1 | 0.5 | 1.7 |
| OU4 | 15.8 | 13.6 | 35.6 | 5.6 | 23.6 | 8.5 | 10.5 | 16.7 | **27.4** | **39.7** | **42.1** | **32.1** | 34.0 | 24.3 | 29.7 | 25.6 | 28.9 | 19.2 | 2.3 |
| EB | 0.3 | 0.4 | 2.6 | 14.6 | 9.3 | 2.7 | 7.0 | 8.7 | 9.4 | 5.2 | 2.8 | 2.2 | 2.3 | 2.3 | 2.0 | 1.2 | 3.1 | 1.3 | 43.0 |
|  | **Ulnar SMA_CC_** | | | | | | | | | | | | | | | | | | |
| BM1 | 0.2 | 1.5 | 0.6 | 12.1 | 0.1 | 13.8 | 12.3 | 2.5 | 2.2 | 0.8 | 0.4 | 1.0 | 1.5 | 0.6 | 0.3 | 0.6 | 2.7 | 1.1 | 5.0 |
| OU1 | 2.8 | 14.3 | 5.7 | **40.1** | 1.2 | 9.5 | 9.0 | 5.2 | 8.3 | 3.9 | 1.1 | 1.4 | 1.6 | 0.9 | 1.2 | 1.8 | 7.1 | 9.2 | 15.5 |
| BM3 | 0.1 | 1.7 | 13.6 | 2.3 | 0.0 | 17.5 | 3.1 | 0.4 | 0.3 | 0.1 | 0.1 | 1.8 | 5.0 | 3.4 | 0.2 | 0.3 | 1.6 | 1.3 | 20.0 |
| OU3 | **68.1** | **50.2** | **25.2** | 2.5 | **62.1** | **31.1** | **44.1** | **60.2** | **58.5** | **72.9** | **80.5** | **72.5** | **67.3** | **74.8** | **76.0** | **75.1** | **56.1** | **59.1** | 0.2 |
| BM4 | 0.3 | 2.5 | 37.8 | 2.4 | 0.0 | 5.1 | 0.8 | 0.1 | 0.1 | 0.0 | 0.0 | 0.4 | 1.0 | 0.9 | 0.1 | 0.1 | 0.4 | 0.3 | **34.9** |
| OU4 | 25.6 | 15.5 | 11.5 | 0.5 | 35.4 | 13.6 | 21.7 | 26.4 | 22.3 | 18.3 | 16.8 | 21.3 | 22.1 | 18.3 | 21.0 | 20.4 | 24.8 | 19.8 | 4.2 |
| EB | 2.8 | 14.3 | 5.7 | 40.1 | 1.2 | 9.5 | 9.0 | 5.2 | 8.3 | 3.9 | 1.1 | 1.4 | 1.6 | 0.9 | 1.2 | 1.8 | 7.1 | 9.2 | 15.5 |

upon each of the four locomotor categories occurring within extant mustelids: fossorial, generalized, natatorial, and scansorial.
