# Supplementary material for: Morphological diversification of biomechanical traits: mustelid locomotor specializations and the macroevolution of long bone cross-sectional morphology
Source: BMC Evol Biol. 2019 Jan 30;19:37. doi: 10.1186/s12862-019-1349-8 (PMC6354431; doi:10.1186/s12862-019-1349-8)
Supplement: Supplementary file 3 — Table S4. Significance and magnitude of alpha values for OU models that were determined to be the best fitting model for humeral cross-sectional traits. Table S5. Significance and magnitude of alpha values for OU models that were determined to be the best fitting model for radial cross-sectional traits. Table S6. Significance and magnitude of alpha values for OU models that were determined to be the best fitting model for ulnar cross-sectional traits. (DOCX 51 kb) [file 12862_2019_1349_MOESM3_ESM.docx]

**Table S4**. Significance and magnitude of alpha values for OU models that were determined to be the best fitting model for humeral cross-sectional traits. ‘Increment’ represents the percentage of bone length from the proximal end at which traits were measured. For alpha, lower and upper confident limits were calculated, and if confidence limits include a value of 0.0, then the estimate of alpha is not considered significant. t represents phylogenetic half life (= ln(2)/ α), and t_Height_, t_Mean_, and t_Min_, represent the phylogenetic half represented as a fraction of tree height, mean branch length, and smallest branch length. These latter three values are to help gauge the strength of alpha relative to the branch lengths of the phylogeny (Cooper et al., 2016).

| **Humeral CSA** | | | | | | | | |
| --- | --- | --- | --- | --- | --- | --- | --- | --- |
| **Increment** | **Model** | **Alpha** | **Lower C.L** | **Upper C.L.** | **t** | **t_Height_** | **t_Mean_** | **t_Min_** |
| 5 | OU3 | 0.22 | -1.621 | 2.060 | 3.163 | 0.1789 | 0.808 | 7.715 |
| **10** | **OU4** | **4.08** | **1.525** | **6.639** | **0.170** | **0.010** | **0.043** | **0.414** |
| 15 | OU4 | 0.57 | -1.043 | 2.186 | 1.213 | 0.068 | 0.310 | 2.958 |
| 20 | OU4 | 1.05 | -0.471 | 2.566 | 0.662 | 0.037 | 0.169 | 1.614 |
| 25 | OU4 | 1.47 | -0.142 | 3.0732 | 0.473 | 0.027 | 0.121 | 1.153 |
| 35 | OU3 | 1.15 | -0.605 | 2.908 | 0.602 | 0.034 | 0.154 | 1.469 |
| **40** | **OU3** | **1.43** | **0.079** | **2.784** | **0.484** | **0.027** | **0.124** | **1.181** |
| **45** | **OU4** | **4.59** | **2.504** | **6.684** | **0.151** | **0.008** | **0.039** | **0.368** |
| **50** | **OU3** | **2.30** | **0.195** | **4.403** | **0.301** | **0.017** | **0.077** | **0.735** |
| **55** | **OU4** | **5.48** | **1.020** | **9.937** | **0.127** | **0.007** | **0.032** | **0.309** |
| **60** | **OU4** | **4.39** | **2.494** | **6.280** | **0.158** | **0.009** | **0.040** | **0.385** |
| **65** | **OU4** | **3.01** | **1.411** | **4.613** | **0.230** | **0.013** | **0.059** | **0.561** |
| 70 | OU4 | 5.01 | -13.852 | 23.876 | 0.138 | 0.008 | 0.035 | 0.337 |
| **75** | **OU4** | **5.38** | **0.560** | **10.203** | **0.129** | **0.007** | **0.033** | **0.314** |
| **80** | **OU4** | **5.90** | **2.635** | **9.163** | **0.118** | **0.007** | **0.030** | **0.287** |
| **90** | **OU4** | **7.96** | **5.138** | **10.781** | **0.087** | **0.005** | **0.022** | **0.212** |
| **Humeral SMA_ML_** | | | | | | | | |
| 5 | OU4 | 0.37 | -1.167 | 1.902 | 1.885 | 0.106 | 0.481338 | 4.598 |
| **10** | **OU4** | **6.71** | **3.128** | **10.297** | **0.1032** | **0.0068** | **0.026** | **0.252** |
| 15 | OU4 | 0.97 | -0.697 | 2.634 | 0.716449 | 0.040 | 0.183 | 1.746 |
| 70 | OU4 | 4.15 | -0.520 | 8.821 | 0.167 | 0.009 | 0.043 | 0.407 |
| 75 | OU4 | 4.11 | -209.991 | 218.205 | 0.169 | 0.009 | 0.043 | 0.412 |
| **80** | **OU4** | **4.620** | **1.071** | **8.168** | **0.150** | **0.008** | **0.038** | **0.366** |
| 90 | OU4 | 1.418 | -0.290 | 3.126 | 0.489 | 0.027 | 0.125 | 1.192 |
| 95 | OU4 | 0.570 | -1.180 | 2.321 | 1.216 | 0.068 | 0.311 | 2.966 |
| **Humeral SMA_CC_** | | | | | | | | |
| 5 | OU3 | 0.34 | -1.159 | 1.823 | 2.067 | 0.116 | 0.528 | 5.042 |
| **10** | **OU4** | **2.29** | **0.401** | **4.185** | **0.302** | **0.017** | **0.077** | **0.737** |
| 15 | OU4 | 0.28 | -1.280 | 1.835 | 2.498 | 0.140 | 0.638 | 6.094 |
| 20 | OU4 | 0.51 | -1.100 | 2.120 | 1.359 | 0.076 | 0.347 | 3.315 |
| 25 | OU4 | 0.55 | -1.142 | 2.248 | 1.254 | 0.070 | 0.320 | 3.059 |
| 30 | OU4 | 0.42 | -1.140 | 1.980 | 1.649 | 0.093 | 0.421 | 4.023 |
| 35 | OU4 | 0.55 | -0.972 | 2.075 | 1.257 | 0.071 | 0.321 | 3.066 |
| 40 | OU4 | 1.34 | -0.265 | 2.938 | 0.519 | 0.029 | 0.132 | 1.265 |
| **45** | **OU4** | **2.32** | **0.716** | **3.924** | **0.299** | **0.017** | **0.076** | **0.729** |
| **50** | **OU4** | **3.11** | **1.517** | **4.705** | **0.223** | **0.013** | **0.057** | **0.543** |
| **55** | **OU4** | **3.48** | **1.608** | **5.347** | **0.199** | **0.011** | **0.051** | **0.486** |
| **60** | **OU4** | **2.41** | **1.042** | **3.786** | **0.287** | **0.016** | **0.073** | **0.700** |
| 65 | OU4 | 1.00 | -0.447 | 2.448 | 0.693 | 0.039 | 0.177 | 1.690 |
| **70** | **OU4** | **2.14** | **0.635** | **3.639** | **0.324** | **0.018** | **0.083** | **0.791** |
| 75 | OU4 | 3.52 | -0.913 | 7.950 | 0.197 | 0.011 | 0.050 | 0.480 |
| **80** | **OU4** | **4.13** | **0.191** | **8.065** | **0.168** | **0.009** | **0.043** | **0.410** |
| **85** | **OU4** | **1.70** | **0.261** | **3.147** | **0.407** | **0.023** | **0.104** | **0.992** |
| **90** | **OU4** | **3.069** | **0.612** | **5.526** | **0.226** | **0.013** | **0.058** | **0.551** |
|  |  |  |  |  |  |  |  |  |

**Table S5**. Significance and magnitude of alpha values for OU models that were determined to be the best fitting model for radial cross-sectional traits. ‘Increment’ represents the percentage of bone length from the proximal end at which traits were measured. For alpha, lower and upper confident limits were calculated, and if confidence limits include a value of 0.0, then the estimate of alpha is not considered significant. t represents phylogenetic half life (= ln(2)/ α), and t_Height_, t_Mean_, and t_Min_, represent the phylogenetic half represented as a fraction of tree height, mean branch length, and smallest branch length. These latter three values are to help gauge the strength of alpha relative to the branch lengths of the phylogeny (Cooper et al., 2016).

| **Radial CSA** | | | | | | | | |
| --- | --- | --- | --- | --- | --- | --- | --- | --- |
| **Increment** | **Model** | **Alpha** | **Lower C.L** | **Upper C.L.** | **t** | **t_Height_** | **t_Mean_** | **t_Min_** |
| 5 | OU4 | 0.34 | -0.954 | 1.642 | 2.014 | 0.113 | 0.514 | 4.911 |
| **15** | **OU3** | **1.56** | **0.221** | **2.901** | **0.444** | **0.025** | **0.113** | **1.083** |
| 25 | OU3 | 3.30 | -184.006 | 190.601 | 0.210 | 0.012 | 0.054 | 0.513 |
| 30 | OU3 | 2.94 | -9.508 | 15.394 | 0.235 | 0.013 | 0.060 | 0.574 |
| 35 | OU3 | 2.69 | -37.217 | 42.599 | 0.258 | 0.014 | 0.066 | 0.628 |
| 40 | OU3 | 2.45 | -0.915 | 5.817 | 0.283 | 0.016 | 0.072 | 0.690 |
| 45 | OU3 | 2.14 | -67.389 | 71.671 | 0.324 | 0.018 | 0.083 | 0.790 |
| 85 | OU3 | 0.54 | -6.139 | 7.227 | 1.274 | 0.072 | 0.325 | 3.107 |
| **90** | **OU3** | **2.47** | **0.241** | **4.704** | **0.280** | **0.016** | **0.072** | **0.684** |
| 95 | OU4 | 0.37 | -0.955 | 1.699 | 1.864 | 0.105 | 0.476 | 4.5464 |
| **Radial SMA_ML_** | | | | | | | | |
| 5 | OU3 | 0.28 | -0.873 | 1.425 | 2.513 | 0.141 | 0.642 | 6.129 |
| 15 | OU3 | 1.39 | -0.733 | 3.512 | 0.499 | 0.028 | 0.127 | 1.217 |
| 30 | OU3 | 2.44 | -1.539 | 6.418 | 0.284 | 0.016 | 0.073 | 0.693 |
| 35 | OU3 | 2.40 | -7.550 | 12.358 | 0.288 | 0.016 | 0.074 | 0.703 |
| 40 | OU3 | 2.16 | -1.114 | 5.431 | 0.321 | 0.018 | 0.082 | 0.783 |
| 45 | OU3 | 2.14 | -12.264 | 16.544 | 0.324 | 0.018 | 0.083 | 0.790 |
| 50 | OU3 | 2.29 | -1.148 | 5.730 | 0.303 | 0.017 | 0.078 | 0.738 |
| 55 | OU3 | 2.37 | -8.453 | 13.192 | 0.293 | 0.016 | 0.075 | 0.713 |
| 60 | OU3 | 2.38 | -0.088 | 4.840 | 0.292 | 0.016 | 0.075 | 0.712 |
| 65 | OU3 | 2.41 | -0.733 | 5.543 | 0.288 | 0.016 | 0.074 | 0.703 |
| 70 | OU3 | 1.91 | -2.418 | 6.239 | 0.363 | 0.020 | 0.093 | 0.885 |
| 75 | OU3 | 1.16 | -1.870 | 4.204 | 0.594 | 0.033 | 0.152 | 1.449 |
| 80 | OU3 | 0.51 | -2.322 | 3.333 | 1.372 | 0.077 | 0.350 | 3.347 |
| 85 | OU3 | 0.58 | -4.868 | 6.022 | 1.201 | 0.067 | 0.307 | 2.929 |
| 90 | OU3 | 2.74 | -4.239 | 9.713 | 0.253 | 0.014 | 0.065 | 0.618 |
| **Radial SMA_CC_** | | | | | | | | |
| 5 | OU3 | 0.30 | -0.898 | 1.496 | 2.319 | 0.130 | 0.592 | 5.656 |
| **25** | **OU3** | **2.19** | **0.119** | **4.270** | **0.316** | **0.018** | **0.081** | **0.770** |
| 30 | OU3 | 2.42 | -0.280 | 5.113 | 0.287 | 0.016 | 0.073 | 0.700 |
| 35 | OU3 | 2.39 | -0.770 | 5.553 | 0.290 | 0.016 | 0.074 | 0.707 |
| 90 | OU3 | 0.37 | -21.546 | 22.277 | 1.899 | 0.107 | 0.485 | 4.631 |

**Table S6**. Significance and magnitude of alpha values for OU models that were determined to be the best fitting model for ulnar cross-sectional traits. ‘Increment’ represents the percentage of bone length from the proximal end at which traits were measured. For alpha, lower and upper confident limits were calculated, and if confidence limits include a value of 0.0, then the estimate of alpha is not considered significant. t represents phylogenetic half life (= ln(2)/ α), and t_Height_, t_Mean_, and t_Min_, represent the phylogenetic half represented as a fraction of tree height, mean branch length, and smallest branch length. These latter three values are to help gauge the strength of alpha relative to the branch lengths of the phylogeny (Cooper et al., 2016).

| **Ulnar CSA** | | | | | | | | | |
| --- | --- | --- | --- | --- | --- | --- | --- | --- | --- |
| **Increment** | **Model** | **Alpha** | **Lower C.L** | | **Upper C.L.** | **t** | **t_Height_** | **t_Mean_** | **t_Min_** |
| **5** | **OU3** | **2.03** | **0.165** | | **3.887** | **0.342** | **0.019** | **0.087** | **0.834** |
| 10 | OU3 | 0.41 | -1.030 | | 1.857 | 1.677 | 0.094 | 0.428 | 4.091 |
| 15 | OU3 | 9.90 | -114189.836 | | 114209.627 | 0.070 | 0.004 | 0.018 | 0.171 |
| 25 | OU3 | 1.98 | -238.297 | | 242.249 | 0.351 | 0.020 | 0.090 | 0.856 |
| 30 | OU3 | 0.80 | -0.789 | | 2.394 | 0.864 | 0.049 | 0.221 | 2.107 |
| 35 | OU3 | 0.54 | -1.029 | | 2.110 | 1.283 | 0.072 | 0.323 | 3.128 |
| 40 | OU3 | 0.59 | -1.179 | | 2.369 | 1.165 | 0.065 | 0.298 | 2.842 |
| 45 | OU3 | 0.64 | -0.931 | | 2.209 | 1.085 | 0.061 | 0.277 | 2.646 |
| 50 | OU3 | 1.11 | -0.532 | | 2.745 | 0.626 | 0.035 | 0.160 | 1.528 |
| 55 | OU3 | 1.00 | -0.404 | | 2.411 | 0.691 | 0.039 | 0.176 | 1.685 |
| 60 | OU3 | 0.82 | -0.651 | | 2.298 | 0.842 | 0.047 | 0.215 | 2.053 |
| **65** | **OU3** | **1.61** | **0.128** | | **3.094** | **0.430** | **0.024** | **0.110** | **1.049** |
| **70** | **OU3** | **1.58** | **0.120** | | **3.045** | **0.438** | **0.025** | **0.112** | **1.068** |
| 75 | OU3 | 2.05 | -1.449 | | 5.554 | 0.338 | 0.019 | 0.086 | 0.824 |
| 80 | OU3 | 2.40 | -71.429 | | 76.236 | 0.288 | 0.016 | 0.074 | 0.703 |
| **85** | **OU3** | **2.10** | **0.024** | | **4.167** | **0.331** | **0.019** | **0.084** | **0.807** |
| 90 | OU3 | 2.93 | -13.708 | | 19.560 | 0.237 | 0.013 | 0.061 | 0.578 |
| 95 | OU1 | 0.10 | -1.1571 | | 1.363 | 6.711 | 0.377 | 1.714 | 16.369 |
| **Ulnar SMA_ML_** | | | | | | | | | |
| 5 | OU3 | 2.40 | | -0.048 | 4.839 | 0.289 | 0.016 | 0.074 | 0.706 |
| 10 | OU3 | 0.52 | | -0.530 | 1.577 | 1.323 | 0.074 | 0.338 | 3.223 |
| 15 | OU3 | 6.79 | | -9494.6342 | 9508.219 | 0.102 | 0.006 | 0.026 | 0.249 |
| 25 | OU3 | 1.52 | | -0.483 | 3.532 | 0.455 | 0.026 | 0.116 | 1.109 |
| 40 | OU3 | 0.30 | | -1.217 | 1.826 | 2.279 | 0.128 | 0.582 | 5.558 |
| 45 | OU4 | 0.49 | | -1.049 | 2.025 | 1.420 | 0.080 | 0.363 | 3.464 |
| 50 | OU4 | 0.98 | | -0.664 | 2.631 | 0.705 | 0.040 | 0.180 | 1.718 |
| 55 | OU4 | 1.11 | | -0.460 | 2.676 | 0.626 | 0.035 | 0.160 | 1.526 |
| 60 | OU4 | 1.54 | | -0.013 | 3.089 | 0.451 | 0.025 | 0.115 | 1.099 |
| 65 | OU3 | 0.81 | | -0.645 | 2.260 | 0.858 | 0.048 | 0.219 | 2.093 |
| 70 | OU3 | 0.57 | | -0.887 | 2.018 | 1.226 | 0.069 | 0.313 | 2.990 |
| 75 | OU3 | 1.02 | | -0.565 | 2.599 | 0.681 | 0.038 | 0.174 | 1.662 |
| 80 | OU3 | 2.09 | | -0.295 | 4.471 | 0.332 | 0.019 | 0.085 | 0.810 |
| 85 | OU3 | 1.53 | | -167.625 | 170.689 | 0.453 | 0.025 | 0.116 | 1.104 |
| 90 | OU3 | 3.59 | | -1.652 | 8.825 | 0.193 | 0.011 | 0.049 | 0.471 |
| 95 | OU1 | 0.20 | | -0.924 | 1.327 | 3.435 | 0.193 | 0.877 | 8.378 |
| **Ulnar SMA_CC_** | | | | | | | | | |
| 5 | OU3 | 8.23 | | -49170.678 | 49187.142 | 0.084 | 0.005 | 0.022 | 0.205 |
| 10 | OU3 | 0.38 | | -1.007 | 1.771 | 1.813 | 0.102 | 0.463 | 4.422 |
| 20 | OU1 | 0.25 | | -1.445 | 1.946 | 2.7674 | 0.156 | 0.706 | 6.748 |
| 25 | OU3 | 0.83 | | -1.130 | 2.785 | 0.837 | 0.047 | 0.214 | 2.043 |
| 30 | OU3 | 0.372 | | -1.459 | 2.190 | 1.896 | 0.107 | 0.484 | 4.625 |
| 35 | OU3 | 0.37 | | -1.381 | 2.129 | 1.853 | 0.10 | 0.473 | 4.520 |
| 40 | OU3 | 1.39 | | -0.966 | 3.740 | 0.500 | 0.028 | 0.128 | 1.219 |
| 45 | OU3 | 3.17 | | -0.157 | 6.505 | 0.218 | 0.012 | 0.056 | 0.533 |
| 50 | OU3 | 4.66 | | -13.584 | 22.898 | 0.149 | 0.008 | 0.038 | 0.363 |
| 55 | OU3 | 4.58 | | -53.637 | 62.798 | 0.151 | 0.009 | 0.039 | 0.369 |
| 60 | OU3 | 2.03 | | -1.064 | 5.125 | 0.341 | 0.019 | 0.087 | 0.833 |
| 65 | OU3 | 1.59 | | -0.1385 | 3.313 | 0.437 | 0.025 | 0.111 | 1.065 |
| 70 | OU3 | 3.79 | | -1.462 | 9.050 | 0.183 | 0.010 | 0.047 | 0.446 |
| 75 | OU3 | 4.94 | | -10.185 | 20.064 | 0.140 | 0.008 | 0.036 | 0.342 |
| 80 | OU3 | 5.74 | | -286.518 | 298.007 | 0.120 | 0.007 | 0.031 | 0.294 |
| 85 | OU3 | 4.91 | | -149.0373 | 158.865 | 0.141 | 0.008 | 0.036 | 0.344 |
| 90 | OU3 | 5.34 | | -73.930 | 84.6895 | 0.12932 | 0.007 | 0.033 | 0.314 |
